# Embedded Micro-detectors for EUV Exposure Control in FinFET CMOS Technology

**DOI:** 10.1186/s11671-021-03645-5

**Published:** 2022-01-05

**Authors:** Chien-Ping Wang, Burn Jeng Lin, Pin-Jiun Wu, Jiaw-Ren Shih, Yue-Der Chih, Jonathan Chang, Chrong Jung Lin, Ya-Chin King

**Affiliations:** 1 Institute of Electronics Engineering, National Tsing Hua University, Hsinchu, Taiwan; 2 Institute of Photonics Technologies, National Tsing Hua University, Hsinchu, Taiwan; 3 National Synchrotron Radiation Research Center, Hsinchu, Taiwan; 4 Design Technology Division, Taiwan Semiconductor Manufacturing Company, Hsinchu, Taiwan

**Keywords:** Extreme ultraviolet (EUV), Detectors, FinFET CMOS technologies

## Abstract

An on-wafer micro-detector for in situ EUV (wavelength of 13.5 nm) detection featuring FinFET CMOS compatibility, 1 T pixel and battery-less sensing is demonstrated. Moreover, the detection results can be written in the in-pixel storage node for days, enabling off-line and non-destructive reading. The high spatial resolution micro-detectors can be used to extract the actual parameters of the incident EUV on wafers, including light intensity, exposure time and energy, key to optimization of lithographic processes in 5 nm FinFET technology and beyond.

## Introduction

Extreme ultraviolet (EUV) is high-energy electromagnetic radiation with wavelengths from 100 to 10 nm [1], which has become a key energy source for many applications. For instance, in the field of solar science, EUV and soft X-Ray (SXR) are used in the solar physics missions at National Aeronautics and Space Administration (NASA) to observe the Sun [2]. On the other hand, EUV microscope captured images of spatial resolution in nanometer scale within a few seconds have been reported [3, 4]. Not to mention in the semiconductor industry, EUV source enables the minimization of the critical dimension (CD) and pushes forward the performance of microchips aggressively. EUV radiation with wavelength of 13.5 nm has become the standard light source in the advanced lithographic systems for integrated circuit (IC) technology nodes beyond 5 nm [5, 6].

To ensure and stabilize the control of CD, the uniformity and consistency of EUV radiation must be kept, which relies on the detecting system for in situ and in-tool EUV monitoring. Conventional silicon-based detecting solutions for EUV radiation use photodiodes [1], which sense EUV light directly by measuring the corresponding photocurrent. As EUV radiation is mostly absorbed within the surface layer of less than 600 nm in most materials [7, 8], extra effort must be taken to ensure good sensitivities. Hence, photodiodes with ultra-shallow junction and defect-free doping are required [1, 9], which in turn increases the complexity in manufacturing and raises barriers for integrating with other devices and circuits.

On the other hand, CMOS Image Sensor (CIS)-based methods employing Active Pixel Sensor (APS) through Backside Illuminated (BSI) technology [10, 11] are also a possible solution to obtain good Quantum Efficiency (QE) and low noise for EUV sensing. Besides CIS-based detectors, Charge-Coupled Device (CCD) is another option for obtaining high resolution and image quality [12]. Yet, all the above technologies require external power supply or batteries installed during sensing, which complicate the in situ sensing design and make them difficult to use in certain environments, for instance, high-vacuum processing chamber and submerging in liquid in emerging lithographic systems.

To meet the recent surge of interest in monitoring in situ EUV intensity distributions without external power supply, an on-wafer EUV micro-detector featuring FinFET CMOS compatibility, compact 1 T pixel structure and in-tool detection is proposed and demonstrated in this work. With this compact 1 T pixel, high spatial resolution array with pixel pitch < 7 μm can be achieved. The proposed embedded micro-detectors can not only provide high spatial resolution; the detected image can be directly written to an in-pixel storage node during exposure without power supply. The stored image can be off-line non-destructive read out, providing timely feedback of on-wafer EUV radiation parameters.

## Methods and Operation Principle

**Fig. 1:**
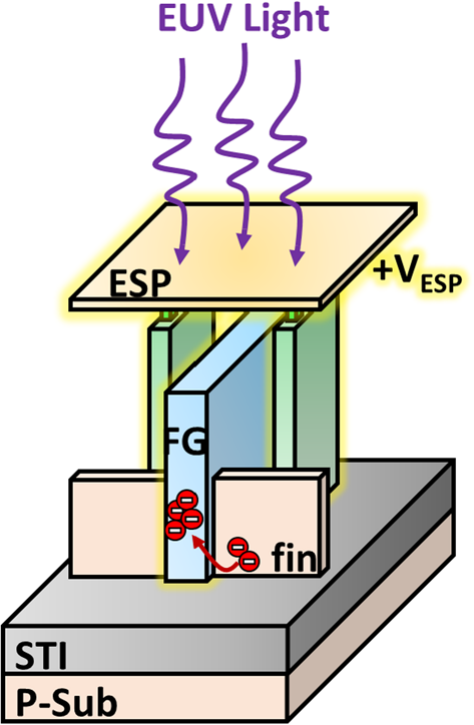
Illustration of the sensing mechanism of the proposed EUV micro-detector

The sensing mechanism and pixel structure of the proposed micro-detector are outlined in Fig. 1. The incident EUV light with wavelength of 13.5 nm is projected on the sensing plane consisting of metal Energy Sensing Pads (ESP). Electrons on these metal ESP get excited and escape from the electrode because of the photoelectric effect, creating positive charged ESP potential (+ *V*_ESP_). This potential will be coupled to the in-pixel storage node, floating gate (FG) through a laterally capacitively coupling structure. When the floating gate potential (*V*_FG_) is high enough, electron’s injection occurs from fin-shape substrate through the thin gate-dielectric layer into FG by the Fowler–Nordheim (FN) tunneling. This then allows the EUV light intensities of each individual pixel to be written onto its corresponding FGs. The amount of FG charge (*Q*_FG_) depends on both the EUV intensity as well as the exposure time, while its level can be read out by off-line wafer level tests. Therefore, the on-wafer micro-detector is proposed to detect and reflect the in situ EUV signal in the advanced lithographic chamber without external power.

The detector’s fabrication process is the same as a standard FinFET CMOS logic process. A brief description is a FinFET process [13–17]. First, the active region is defined by mask and forms a fin-shape substrate. Second, Sallow Trench Isolation (STI) is used to isolate the devices. Then, oxidation will be performed to form thin dielectric. Next, polysilicon is deposited and defined as gate region. And the Lightly Doped Drain (LDD) will be implanted to reduce hot carrier injection and other non-ideal effects. After LDD, Spacer and Source (SL)/Drain (BL) region is formed. Then, gate material is replaced with metal (FG) for better reliability. Finally, back-end-of-line (BEOL) process is used to interconnect and construct the coupling structure of the detector (RS&ESP).

**Fig. 2:**
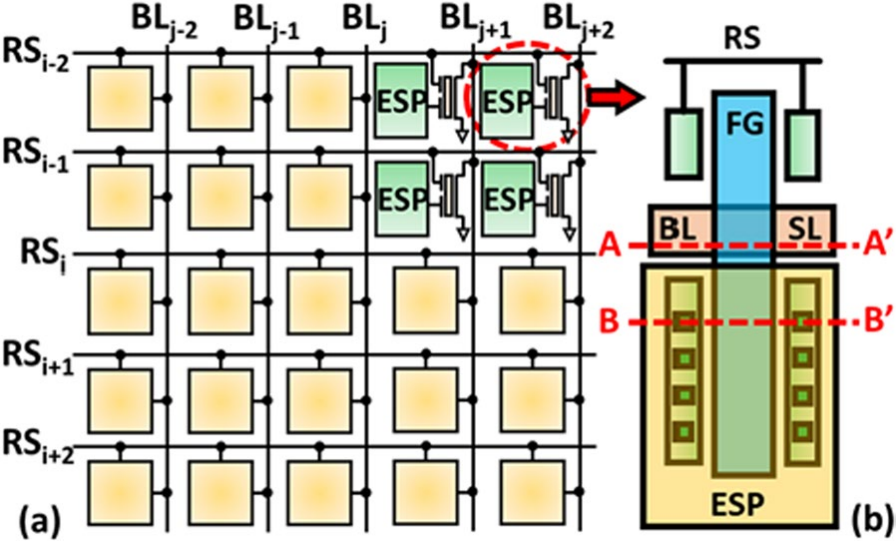
**a** The schematic of the proposed high-density micro-detectors and **b** the top-view layout of the 1 T pixel consist of a single floating gate transistor

The schematic and pixel layout of micro-detectors are shown in Fig. 2a, b, respectively. A pixel is composed of one n-channel FG transistor with two coupling gates. One of the coupling gates is connected to Row-Select (RS) line, which is responsible for signal control during readout; the other coupling gate is connected to ESP, consisting of Cu-based metal for EUV sensing. The filled factor of the proposed pixel determined by the ratio of ESP area against the overall pixel area is around 30%.

**Fig. 3:**
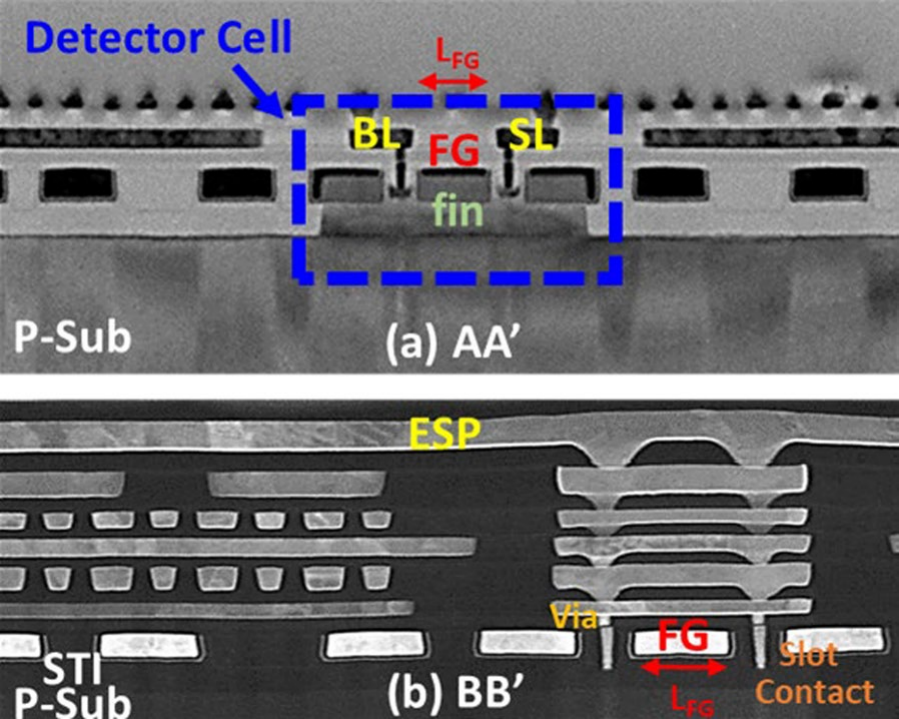
TEM image of the cross section of **a** the n-channel FG FinFET transistor and **b** the ESP with a slot-contact coupling structure, where the FG length, *L*_FG_ = 0.14 μm

The cross section view of the pixel is shown in the Transmission Electron Microscope (TEM) image along AA’ line in Fig. 3a. On the other hand, the coupling structure of RS and ESP realized by closely placed slot contacts next to FG can be further observed in the TEM image along BB’ line in Fig. 3b; hence, *V*_FG_ is determined by the potential of RS (*V*_RS_) and *V*_ESP_ through these laterally coupling structure.

## Experimental Results and Discussion

### Optical Simulation and Detectors Modeling

**Fig. 4:**
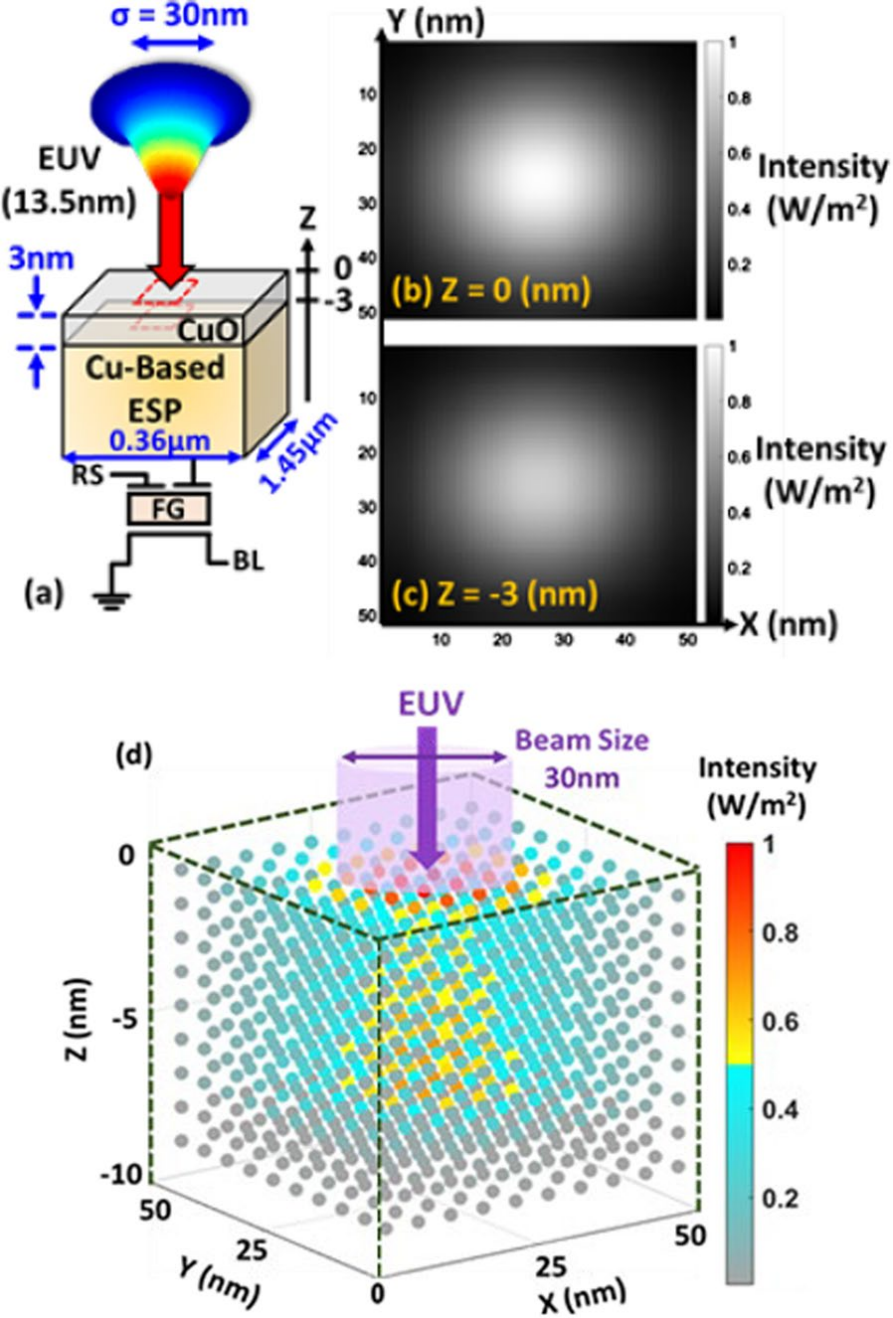
**a** The FDTD simulation setup and the 2D intensity distribution at **b** *Z* = 0 nm and **c** *Z* = − 3 nm. **d** The 3D intensity distribution, indicating the energy profile of the injected EUV light

It is known that EUV light can be absorbed by a thin layer of most materials [7, 8], which makes the EUV detector design much more challenging. Here, optical simulation by Finite-Difference Time-Domain (FDTD) is firstly used to estimate the EUV intensity profile on ESP to facilitate the pixel design. By using this simulation tool, nanoscale optical device can be precisely modeled by resolving the Maxwell’s equations on a mesh in the time domain [18]. The incident EUV light intensity is set to be Gaussian distribution with standard deviation of 30 nm in space, projecting on the Copper ESP covered by native Copper oxide (CuO) of 3 nm [19], as illustrated in Fig. 4a. The FDTD optical simulation parameters of each dielectric films with film thickness < 10 nm at wavelength of 13.5 nm are summarized in Table 1. From the simulation results in Fig. 4b, c, the 2D distribution at the interface of Vacuum/CuO (*Z* = 0 nm) and CuO/Cu (*Z* = − 3 nm) indicates that a significant amount of incident EUV is absorbed by native CuO. Furthermore, little spread is found in the dielectric film, indicating the direct absorption of EUV light dominates, while scattering effect is limited in these oxide layer covered on ESP structures. Data show that native CuO will cause ~ 19% loss in signal, as shown in the 3D distribution in Fig. 4d.

**Table 1:** FDTD parameters at wavelength of 13.5 nm

Dielectric	Refractive index	Extinction coefficient	REF
Copper oxide (CuO)	0.948	0.072	[20]
Silicon dioxide (SiO_2_)	0.97352	0.01608	[21]
Aluminum oxide (Al_2_O_3_)	0.9714	0.035	[22]

**Fig. 5:**
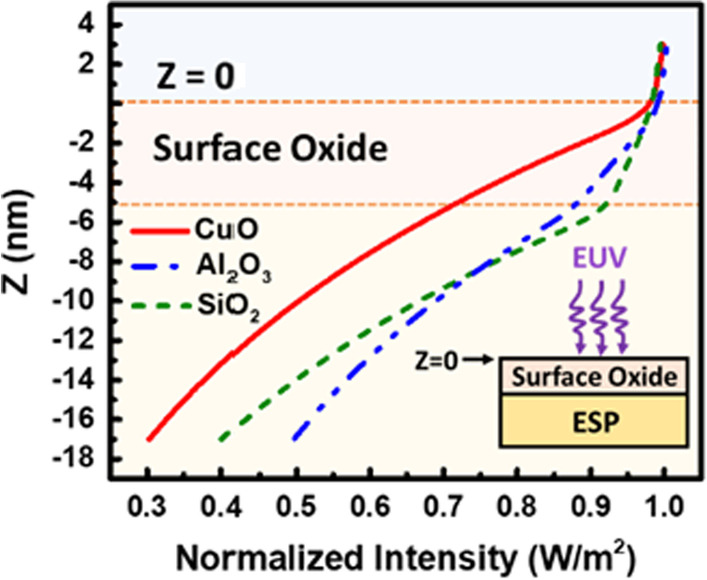
The simulated EUV intensity profile along the *Z*-axis as it penetrates through the metal ESP covered by 5-nm-thick oxides

The intensity profile of depth (along the *Z*-axis) in different surface oxide and Cu-based ESP reveals that surface oxide layer can critically affect the EUV signal reaching ESP, as shown in Fig. 5. It is found that CuO can significantly block EUV light with even a thickness less than 5 nm.

**Fig. 6:**
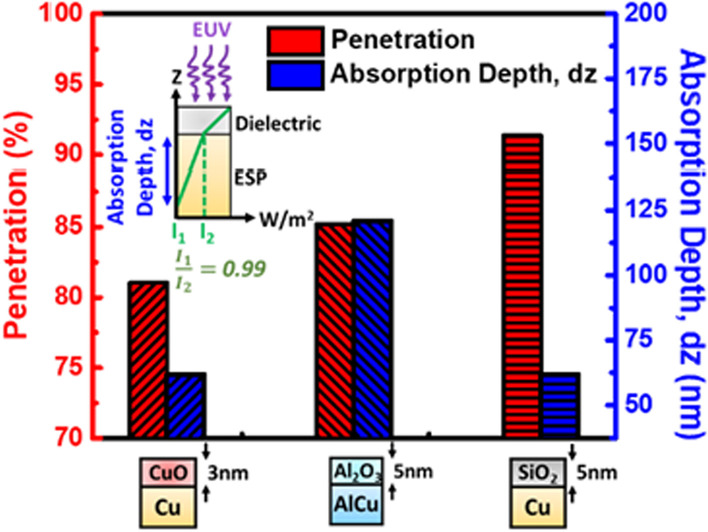
EUV penetrated percentage through oxide and ESP absorption depth of different oxide/metal compositions

Here, the impact of native aluminum oxide (Al_2_O_3_) with thickness of 5 nm [23] on AlCu-based (Aluminum Copper alloy) ESP is also considered. The penetration ratio and absorption depth of the three types of ESP/oxide stacks compared in Fig. 6 suggest that replacing CuO by SiO_2_ can enhance EUV penetration by 10%, while the thickness of AlCu-based ESP needs to be above 150 nm to ensure EUV absorption. The further performance comparison based on measurement data between Cu-based ESP and AlCu-based ESP detectors will be addressed in our future work.

Next, the photo-response of the Cu-based ESP is measured and quantified through specially designed tests. The photography of the experimental setup uses synchrotron radiation of 13.5 nm, including the proposed micro-detectors, as well as a real-time EUV photocurrent (*I*_ph_) measurement by a wireless module. During this experiment, the exposure time is controlled by an electrical shutter with switching time in millisecond level, while the light intensity is monitored simultaneously by a power meter. The measured photo-response current in real-time waveform can be used to check for alignment of the light beam.

**Fig. 7:**
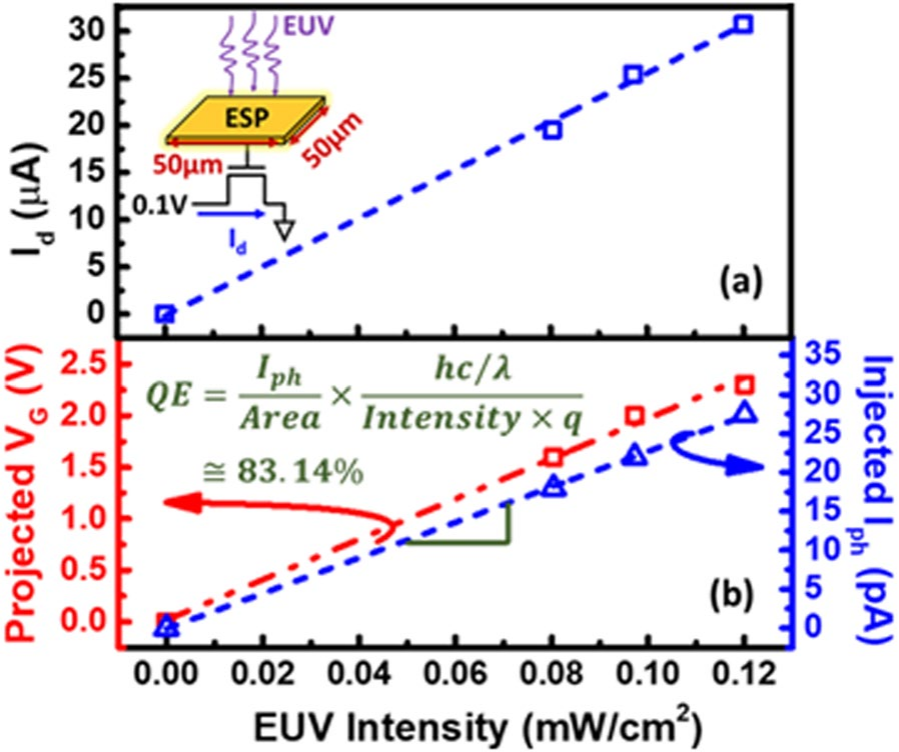
The **a** measured data and **b** the photocurrent response induced by EUV light, projected by this transistor

To estimate the Quantum Efficiency (QE) of ESP, the real-time EUV *I*_ph_ is firstly measured by the channel current of a n-channel MOSFET with an AlCu-based ESP connected to its gate. Its ESP is charged under EUV exposure, turning on the transistor and raising its channel current (*I*_d_), as demonstrated in Fig. 7a.

In this study, QE is essentially defined as followed:

1 $${\text{QE}} = \frac{{I_{{{\text{ph}}}} }}{{{\text{Sensing}}\;{\text{Area}}}} \times \frac{{{\text{Photon}}\; {\text{Energy}}}}{{{\text{EUV}}\;{\text{Intensity}} \times q}},$$

where *q* is the elementary electric charge.

Therefore, QE of the surface ESP is estimated to be 83.14% by injecting a gate current which fits the channel current under a certain light intensity, as shown in Fig. 7b. In other words, 83.14% of incident photons will be converted to electrons on ESP due to the photoelectric effect, leading to positively charged ESP. The measured QE can be affected significantly by multiple non-ideal effects, for instance, absorption of native oxide, surface traps and so forth [24–26]. On the other hand, QE of conventional silicon-based EUV sensors is estimated as the amount of generated photoelectron divided by the incident photons, which is generally determined by its photoconductivity gain, biasing conditions and surface/interface quality, etc. [27–29].

**Fig. 8:**
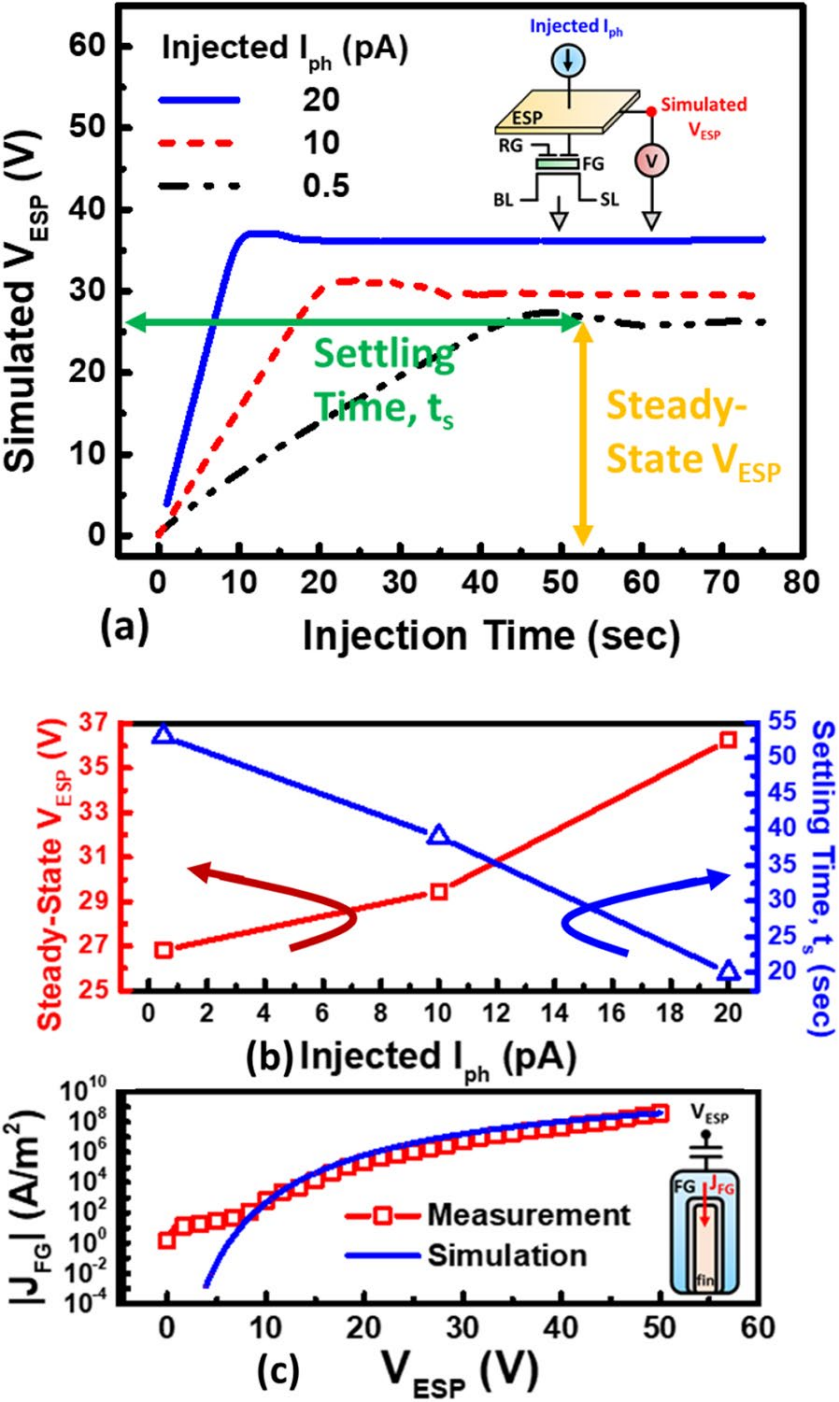
**a** The transient response of simulated ESP potential under increasing injected *I*_ph_. And **b** the steady-state *V*_ESP_ and the settling time under different *I*_ph_ injection, and **c** measured and simulated FG tunneling current density (*J*_FG_) induced by ESP potential

Furthermore, the simulated transient response of *V*_ESP_ with the corresponding injected *I*_ph_ is compared in Fig. 8a, both the settling time (*t*_s_) and the steady-state *V*_ESP_ can be obtained in Fig. 8b. As the data indicated, the settling time of ESP is about 50 s; hence, the minimum exposure time is set to be 500 s in this study to ensure the stability of EUV light. In an actual EUV scanner at the level of a few W/cm^2^, the settling time is expected to be less than 1 μs, while the exposure time of our on-wafer detector is expected to be higher than 20 μs. The injection current density into FG (*J*_FG_) induced by *V*_ESP_ is further shown in Fig. 8c, indicating higher *V*_ESP_ results in higher *J*_FG_ within a period of exposure time. These enable the model between the stored *Q*_FG_ and EUV intensity to be established.

### EUV Detection Results

As discussed in Sect. 2, random charge may be stored in FG during the plasma process of manufacturing; therefore, initial calibration procedure is needed before EUV exposure. The threshold voltage (*V*_th_) distribution converged to its neutral state (*V*_th_ at 4 V). It is done by biasing RS or substrate to high voltage so that channel hot carrier injection or FN tunneling may occur to clear out the initial *Q*_FG_.

**Fig. 9:**
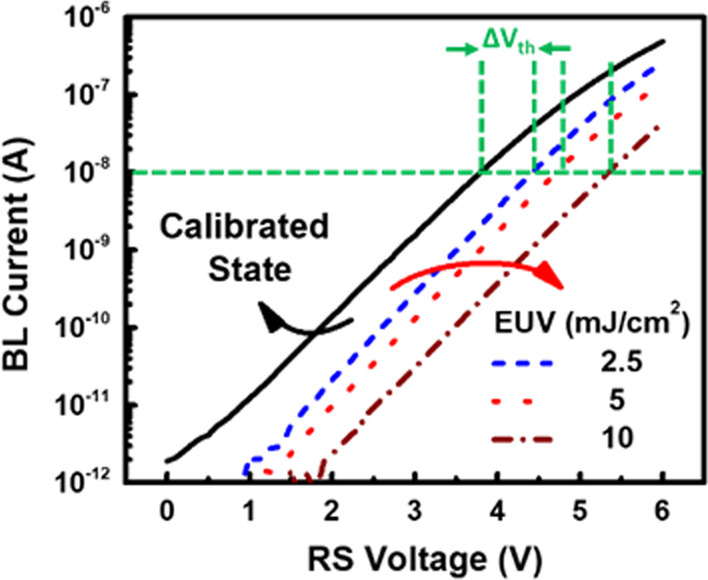
The measured IV characteristics of a discrete micro-detector with increasing amount of EUV energy applied

The electrical characteristics of the proposed micro-detector before and after EUV exposure are compared in Fig. 9. Under the fixed intensity of 5 μW/cm^2^, as the exposure time as well as the exposed EUV energy increases, tunneling current over a longer period of time is expected to occur between FG and substrate, leading to more FG charge. Hence, the IV curve shifts further to the right with increasing amount of FG charge, as reflected by the shift in *V*_th_, as indicated in this plot. For monitoring EUV level during wafer-level test, *V*_th_ extraction can be done externally through automatic testing programs, measuring BL current under *V*_RS_ sweeps. To increase readout speed, readout circuits such as a *V*_th_ extractor circuit [30] can be also incorporated through FinFET platform.

**Fig. 10:**
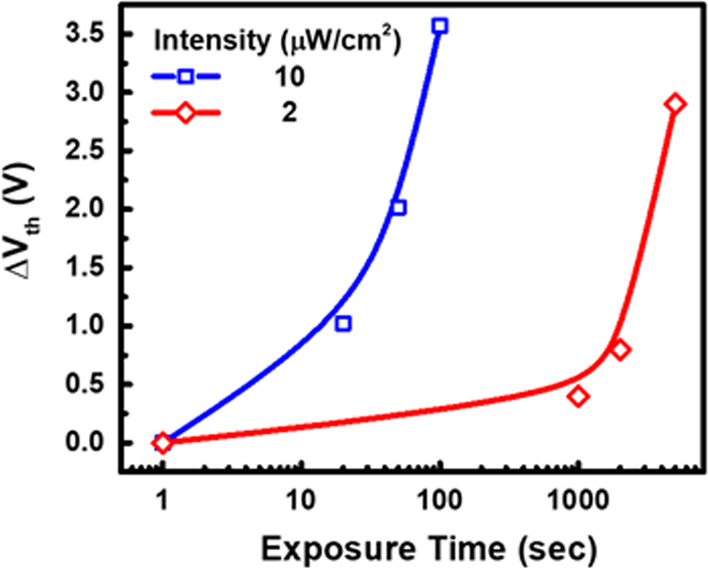
The photo-response of the micro-detector with exposure time under different EUV intensity settings

The measured *V*_th_ shift (∆*V*_th_) can reflect the projected EUV intensity and exposure time, as indicated in Fig. 10. As the EUV intensity increases, the photocurrent level on ESP is expected to raise, resulting in higher ESP potential. Consequently, more charge will be recorded in the in-pixel storage node (FG). Therefore, the combination of EUV light intensity and exposure time will determine the final *Q*_FG_, which in turn reflects on the readout ∆*V*_th_. On the other hand, if the light intensity is too low, the detector will not be able to register the response as FG charge when tunneling effect is minimal even with long exposure time. In contrast, under high enough light intensity, the amount of FG charge is expected to be proportional to exposure time.

**Fig. 11:**
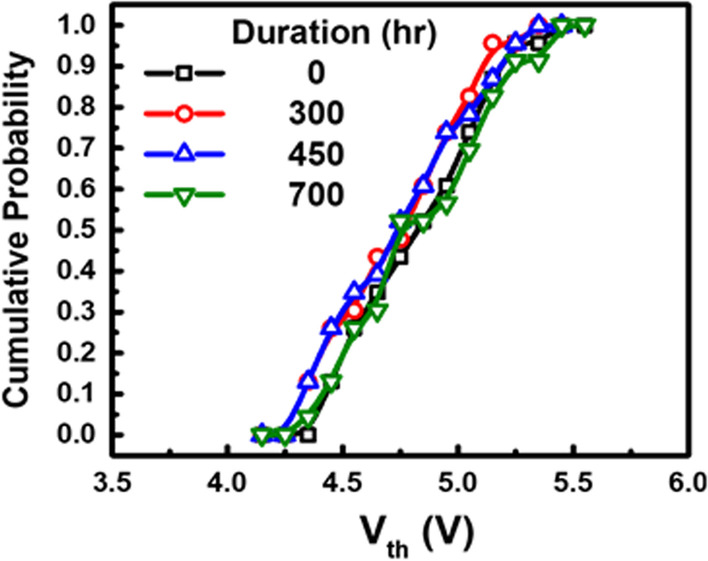
The data integrity of the stored detection results of 5 mJ/cm^2^ at room temperature, enabling off-line automatic Wafer Acceptance Test (WAT) readings

One of the unique features of the proposed micro-detectors is its capability to store and record the detected image without external power supply, as demonstrated in Fig. 11. The detected EUV image can be recorded in the in-pixel FG for months in room temperature after exposure, enabling follow-up off-line and non-destructive electrical reading.

## Conclusions

In this work, a novel embedded micro-detector for in situ and in-tool EUV imaging featuring FinFET CMOS compatibility, compact pixel structure and high spatial resolution is demonstrated. The promising EUV micro-detectors can provide robust detection, precise monitoring for battery-less detection in EUV chambers.

## Data Availability

Not applicable.

## Abbreviations

AlCu: Aluminum Copper Alloy
Al_2_O_3_: Aluminum oxide
APS: Active Pixel Sensor
BEOL: Back-end-of-line
BL: Bit line
BSI: Backside illuminated
CCD: Charge-coupled device
CD: Critical dimension
CIS: CMOS Image Sensor
CMOS: Complementary Metal Oxide Semiconductor
Cu: Copper
CuO: Copper oxide
ESP: Energy Sensing Pad
EUV: Extreme ultraviolet
FDTD: Finite-Difference Time-Domain
FG: Floating gate
FinFET: Fin Field-Effect Transistor
FN: Fowler–Nordheim tunneling effect
IC: Integrated circuit
I_ph_: Photocurrent
J_FG_: FG current density
LDD: Lightly Doped Drain
NASA: National Aeronautics and Space Administration
QE: Quantum Efficiency
Q_FG_: FG charge
RS: Row select
SiO_2_: Silicon dioxide
SL: Source line
STI: Sallow Trench Isolation
SXR: Soft X-ray
TEM: Transmission Electron Microscope
t_s_: Settling time
V_ESP_: Energy Sensing Pad Potential
V_FG_: Floating gate potential
V_RS_: Row-select potential
V_th_: Threshold voltage
WAT: Wafer Acceptance Test

## References

[1] Shi L, Nihtianov S (2012). Comparative study of silicon-based ultraviolet photodetectors. IEEE Sens J.

[2] Young PR (2021). Future prospects for solar EUV and soft X-ray spectroscopy missions. Front Astron Space Sci.

[3] Wachulak PW, Torrisi A, Bartnik A (2017). A desktop extreme ultraviolet microscope based on a compact laser-plasma light source. Appl Phys B.

[4] Gunjala G, Wojdyla A, Sherwin S (2020). Extreme ultraviolet microscope characterization using photomask surface roughness. Sci Rep.

[5] Turkot B, Carson S, Lio A (2017) Continuing Moore's law with EUV lithography. In: 2017 IEEE international electron devices meeting (IEDM), 2017, pp 14.4.1–14.4.3

[6] Xie R et al. (2016) A 7 nm FinFET technology featuring EUV patterning and dual strained high mobility channels. In: 2016 IEEE international electron devices meeting (IEDM), pp 2.7.1–2.7.4

[7] Luong V, Philipsen V, Hendrickx E, Opsomer K, Detavernier C, Laubis C, Scholze F, Heyns M (2018). Ni-Al alloys as alternative EUV mask absorber. Appl Sci.

[8] Kang HY, Hwangbo CK (2009). Absorber stack with transparent conductive oxide layer for extreme ultraviolet lithography. J Vac Sci Technol B Microelectron Nanometer Struct Process Meas Phenom.

[9] Shi L, Nanver LK, Nihtianov SN (2011) Stability characterization of high-sensitivity silicon-based EUV photodiodes in a detrimental industrial environment. In: IECON 2011—37th annual conference of the IEEE industrial electronics society, 2011, pp 2651–2656

[10] Rao PR, Laubis C, Nihtianov S (2014) Backside illuminated CMOS image sensors for extreme ultraviolet applications. In: Sensors, 2014 IEEE, pp 1660–1663

[11] Stern RA, Shing L, Waltham N, Mapson-Menard H, Harris A, Pool P (2011). EUV and soft X-ray quantum efficiency measurements of a thinned back-illuminated CMOS active pixel sensor. IEEE Electron Device Lett.

[12] Shang Y, Guan Y, Liu Y, Zhao X (2008) The design of EUV CCD camera. In: Proc. SPIE 7021, high energy, optical, and infrared detectors for astronomy III, p 70211O

[13] Okano K et al. (2005) Process integration technology and device characteristics of CMOS FinFET on bulk silicon substrate with sub-10 nm fin width and 20 nm gate length. In: 2005 IEEE international electron devices meeting (IEDM), pp 721–724

[14] Veloso A, Lee JW, Simoen E, Ragnarsson L-Å, Arimura H, Cho MJ, Boccardi G, Thean A, Horiguchi N (2014). Replacement metal gate/high-k last technology for aggressively scaled planar and FinFET-based devices. ECS Trans.

[15] Standaert T et al. (2016) BEOL process integration for the 7 nm technology node. In: 2016 IEEE international interconnect technology conference/advanced metallization conference (IITC/AMC), pp 2–4

[16] Han X, Yang C, Li D, Zhang S (2008) A simple nano-scale patterning technology for FinFET fabrication. In: 2008 9th international conference on solid-state and integrated-circuit technology, pp 1340–1342

[17] Tanwar K, Canaperi D, Lofaro M, Tseng W-T, Patlolla R, Penny C, Waskiewicz C (2013). BEOL Cu CMP process evaluation for advanced technology nodes. J Electrochem Soc.

[18] Ishak AM, Ishak MT, Jusoh MT, Syed Dardin SF, Judd MD (2017). Design and optimization of UHF partial discharge sensors using FDTD modeling. IEEE Sens J.

[19] Kim SJ, Kim S, Lee J (2021). Color of copper/copper oxide. Adv Mater.

[20] Brimhall N, Herrick N, Allred DD, Turley RS, Ware M, Peatross J (2009). Measured optical constants of copper from 10 nm to 35 nm. Opt Express.

[21] Bodermann B, Wurm M, Diener A, Scholze F, Groß H (2009) EUV and DUV scatterometry for CD and edge profile metrology on EUV masks. In: Proc. SPIE, vol 7470, p 74700F

[22] Rebellato J, Meltchakov E, Soufli R, Rossi SD, Zhang X, Auchère F, Delmotte F (2018) Analyses of tabulated optical constants for thin films in the EUV range and application to solar physics multilayer coatings. In: Proc. SPIE, vol 10691, p 106911U

[23] Saif MTA, Zhang S, Haque A, Hsi KJ (2002). Effect of native Al_2_O_3_ on the elastic response of nanoscale Al films. Acta Mater.

[24] Schuster J, Bellotti E (2014). Evaluation of quantum efficiency, crosstalk, and surface recombination in HgCdTe photon-trapping structures. J Electron Mater.

[25] Booker RL, Geist JC (1982). Photodiode quantum efficiency enhancement at 365 nm: optical and electrical. Appl Opt.

[26] Hamden ET, Jewell AD, Shapiro CA, Cheng SR, Goodsall TM, Hennessy J, Hoenk ME, Jones T, Gordon S, Ong HR, Schiminovich D, Martin DC, Nikzad S (2016). Charge-coupled devices detectors with high quantum efficiency at UV wavelengths. J Astron Telesc Instrum Syst.

[27] Mudgal S, Singh S, Komarala VK (2018) Interfacial spectral response under voltage and light bias to analyse low voltage in amorphous-crystalline silicon heterojunction solar cell with S-shape characteristics. In: 2018 IEEE 7th world conference on photovoltaic energy conversion (WCPEC) (a joint conference of 45th IEEE PVSC, 28th PVSEC & 34th EU PVSEC), pp 2158–2161

[28] Li X, Carey JE, Sickler JW, Pralle MU, Palsule C, Vineis CJ (2012). Silicon photodiodes with high photoconductive gain at room temperature. Opt Express.

[29] Kuroda R, Kawada S, Nasuno S, Nakazawa T, Koda Y, Hanzawa K, Sugawa S (2014). A highly ultraviolet light sensitive and highly robust image sensor technology based on flattened Si surface. ITE Trans Media Technol Appl.

[30] Johnson MG (1993). An input-free VT extractor circuit using a two-transistor differential amplifier. IEEE J Solid-State Circuits.

